# Human umbilical cord-derived mesenchymal stem cells alleviate valproate-induced immune stress and social deficiency in rats

**DOI:** 10.3389/fpsyt.2024.1431689

**Published:** 2024-08-22

**Authors:** Shixiong Sun, Shilin Luo, Jie Chen, Ou Zhang, Qiongying Wu, Nianju Zeng, Jinlian Bi, Chunbing Zheng, Tenglong Yan, Zhiyuan Li, Jindong Chen, Yilei Zhang, Bing Lang

**Affiliations:** ^1^ Department of Psychiatry, National Clinical Research Center for Mental Disorders, and National Center for Mental Disorders, The Second Xiangya Hospital of Central South University, Changsha, Hunan, China; ^2^ Department of Neurology, Xiangya Hospital of Central South University, Changsha, China; ^3^ Department of Rehabilitation, Xiangya Boai Rehabilitation Hospital, Changsha, Hunan, China; ^4^ Changsha Institute of Industrial Technology for Stem Cell and Regenerative Medicine, Hunan Yuanpin Cell Technology Co. Ltd. (Yuanpin Biotech), Changsha, Hunan, China

**Keywords:** autism spectrum disorders, human umbilical cord-derived mesenchymal stem cells, microglia, valproic acid, immune stress

## Abstract

**Introduction:**

Autism spectrum disorders (ASD) are a set of heterogeneous neurodevelopmental disorders characterized by impaired social interactions and stereotypic behaviors. Current clinical care is palliative at the most and there remains huge unmet medical need to fully address the core symptoms of ASD. Human umbilical cord-derived mesenchymal stem cells (hUC-MSCs) are emerging as a promising candidate for ASD treatment, but the precise mechanism remains controversial.

**Methods:**

In vitro studies we performed the transwell migration assay to explore the interaction between hUC-MSCs and the primary-cultured cortical neurons. Then we determined the therapeutic effects of intravenous administration of hUC-MSCs in rats challenged with valproic acid (VPA) during gestation, a well-defined rat model of autism.

**Results:**

Our studies showed that hUC-MSCs promoted the growth of primary-cultured cortical neurons. Furthermore, our results demonstrated that hUC-MSCs significantly alleviated microglial activation in the brain, especially in the anterior cingulate cortex, and effectively improved the sociability of the VPA-exposed rats.

**Discussion:**

These results offer valuable insights for clinical translation and further research on the mechanisms of hUC-MSCs in psychiatric disorders characterized by microglial activation, particularly in cases of autism, shall be warranted.

## 1 Introduction

Autism spectrum disorders (ASD) are known as a group of severe neurodevelopmental disabilities with multifaceted manifestation. The core symptoms of ASD consist of several main symptoms: impairment in communication skills, meaningless repetitive behavior, and speech disorder (1). Based on recent epidemiological data (2), ASD is rising rapidly due to advancements in clinical evaluation, screening, and diagnosis. The average prevalence of ASD in Asia, Europe, and North America is approximately 1%. Neurodevelopmental abnormalities are believed to be the primary factor contributing to ASD, whilst other factors like immune dysfunction, inflammation, abnormal metabolism, and environmental stress also facilitate the occurrence of ASD. As a result, current treatments are mostly against the adjunct symptoms of ASD such as anxiety, depression, or attention deficits, and the core features of ASD remain largely unaddressed.

Microglia is the predominant immune cells resident in the central nervous system which makes up approximately 5-15% of brain cells (3). Amounting evidence has shown that they are closely involved in synaptogenesis and immunomodulation through their unique abilities in synaptic pruning and cytokine production (4, 5). Recently, it has been widely appreciated that microglia may contribute to the pathology of ASD through over-activated synaptic pruning and complement-mediated microglial phagocytosis (6) as increased activation of microglia and astroglia has been reported in hippocampus, cerebellum, and prefrontal cortex during early developmental stages of ASD (7, 8). Animal model studies have also indicated that cortical microglia in animals exposed to valproic acid (VPA) are functionally blunt to neuronal signals and can exacerbate the activity of reactive astrocytes (7). Such dysfunction can frequently cause abnormal immune stress such as high levels of pro-inflammatory cytokines, abnormal activation of T-cell subsets and even auto-antibodies which are always the endophenotypes of ASD rodent models or human cases. Therefore, microglia play a critical role in the pathophysiology of ASD and can be harnessed to alleviate ASD core symptoms.

Mesenchymal stem cells (MSCs) have long been proposed in regenerative medicine and tested in clinical trials (9). However, recent studies have demonstrated that they also have a significant impact on the proliferation, migration and differentiation of microglia, thus orchestrating brain development and immunomodulation (10–12). Compared with bone-marrow (or other origin) derived MSCs, human umbilical cord-derived mesenchymal stem cells (hUC-MSCs) possess unique advantages such as easy availability (no risk of invasion procedure, anesthesia, pain or infection), faster proliferation and the excellent ability of immunomodulation. This suggests that hUC-MSCs may be beneficial in treating conditions with immune dysfunction including ASD (13). Indeed, hUC-MSCs can secrete anti-inflammatory cytokines such as IFN-α and exhibit low immunogenic profile with absence of co-stimulatory molecules and HLA-DR together with very low expression of HLA class I (14). Therefore, hUC-MSCs have been applied in multiple clinical trials such as diabetes type I, SLE, multiple sclerosis, allograft-related diseases, and even severe acute respiratory syndrome caused by the coronavirus type 2 et al, and showed good tolerance, safety and treatment efficacy (15–19). Notably, in a recent clinical trial (20), half of the participants (6 out of 12 ASD cases) who received intravenous hUC-MSCs infusion displayed improved core symptoms of ASD, highlighting encouraging therapeutic effects with unknown mechanisms.

Although several clinical trials which utilize hUC-MSCs to treat neurological conditions including autism (20, 21) have been recently published with promising results, the underpinning mechanisms are so far inconsistent and ambiguous which greatly limit the application of hUC-MSCs in clinical practice despite of the huge unmet medical demands. In the present study, utilizing rats challenged with valproic at the gestation day 13, a well-established human autism model, we determined the therapeutic effects of hUC-MSCs at different dosages for the aberrant cognitive manifestation reminiscent of the core symptoms of human ASD in rats. We found that hUC-MSCs effectively improved the cognitive presentation of the rats which may partially attribute to the alleviated activation of microglia in the cortex especially the anterior cingulate cortex (ACC). Our data further support the hypothesis that an orchestrated immune modulatory capacity may underpin the therapeutic potential of hUC-MSCs for human autism.

## 2 Materials and methods

### 2.1 Animals and drug administration

Time-mated breeding pairs of Sprague Dawley rats were set up and the day with the appearance of white plug in the vaginal opening of females was deemed as embryonic day 0.5 (E0.5). These pregnant rats were raised alone and received a single dose of 400mg/kg of VPA injection intraperitoneally (VPA Group, Sigma P4543, dissolved in sterilized saline) or sterilized saline (Control group) on the day of E13. 5. For the treatment experiment of VPA-treated offspring, tail deformity was a prerequisite. Male offspring were randomly divided into categories of vehicle, low-dosage, and high-dosage (each contains 6 rats) at postnatal day 21. hUC-MSCs were obtained from Yuanpin Cell Biotechnology Group Co., Ltd (http://www.yuanpinbio.com) with cell viability and toxicology monitored. Different dosages of hUC-MSCs (1 X 10^6^/kg or 3X10^6^/kg) were administered via tail vein at postnatal day 60. The vehicle group received filtered culture medium of hUC-MSCs. All the animals were housed at 22 ± 2 °C temperature, 60± 5% relative humidity, 12 h light/dark cycle (lights on at 7:00 AM) with access to food and water ad libitum. This study has been approved by the Animal Ethics Committee (Approval number 2022711) and all animal procedures were performed in agreement with the guidelines of Guidelines and Regulation of Laboratory Animals Used for Biomedical Studies of the Xiangya Second Hospital, Central South University, Changsha, China.

### 2.2 Tissue collection

Upon the completion of all the behavioral tests, rats were anesthetized with over-dose pentobarbital (0.2% m/w, 0.2 ml/100g) and perfused with cold phosphate buffer saline (PBS) followed by 4% paraformaldehyde. Then their brains were removed and postfixed in 4% paraformaldehyde for 6 h, after which they were transferred into 30% sucrose for 24 h, and 30% sucrose were refreshed every 24h until the brain sank. Finally, the brains were embedded in O.C.T and stored at -80°C.

### 2.3 Immunofluorescence

Immunofluorescent staining was performed as previously reported (22). Rat brains were sectioned into 45-μm-thick coronal slices. Brain slices were blocked with 1% bovine serum albumin for 60 minutes at room temperature. Slices were incubated overnight with Iba1 antibody (1:500, FUJIFILM Wako Pure Chemical Corporation Cat# 011-27991, RRID: AB_2935833) at 4°C and rinsed in PBS. Then, they were incubated for 1h at room temperature with the corresponding secondary antibody (Alexa Fluor^®^ 488, Abcam Cat# ab150077, RRID: AB_2630356). The staining of nuclei was performed using double-stranded DNA staining (DAPI) included in mounting medium (Abcam, ab104139).

### 2.4 Behavioral tests

The behavioral tests’ equipment was purchased from Clever Sys Inc, and the recorded videos were analyzed quantitatively using TopScan behavior analysis system.

#### 2.4.1 Open field test

The test was carried out at postnatal day 60 and postnatal day 90 (1 month later MSCs mainline). The open field test was performed in a 100 × 100 cm square with 50 cm high walls. This square was divided into two parts zones: 50 × 50 cm central square and other zone of the total square. Rats were placed in the center of the square and recorded for 5 minutes. The distance and time that rats spent exploring the two parts zones would be measured. 75% ethanol were used to thoroughly cleaned the open field after every single test so that odor interference could be avoided.

#### 2.4.2 Three-chamber social interaction test

This test was performed in a rectangular three-chamber box, with two lateral chambers connected to a central chamber. All chambers have the same shape and size (30 l × 35 w× 35 h cm), and each lateral chamber were symmetrically placed a small metal cage on the corner. The complete test process contained three stages. Every rat was placed in the central chamber to explore freely for 10 minutes in each stage, and there was a 24-hour interval between stages. In the first stage, no rats were placed in the small metal cage. The test rat could explore the box for habituation. Then, an unacquainted SD rat was placed in one of the two small metal cages in the next stage, and another unacquainted SD rat was put in the other small metal cage in the third stage. The time the test rat spent sniffing at the unfamiliar was measured to indicate social interaction trends. Similarly, those chambers and metal cages were thoroughly cleaned with 75% ethanol after each rat explored.

### 2.5 Cell culture

Pregnant female rats at embryonic 14.5 days (E14.5) were anesthetized with CO_2_. After the uterus was exposed, the embryos were harvested and fully washed with pre-chilled Hanks Buffered Saline Solution (HBSS) containing 0.27 mM pyruvate and a 1× antibiotic mixture (100 units/mL penicillin G, 0.25 g/mL amphotericin B, and 100 units/mL streptomycin, Gibco). Brains were dissected out with the connective tissue carefully removed under a microscope. The cerebral cortex was collected and digested in HBSS with 0.25% trypsin and 0.2 mg/ml deoxyribonuclease (Gibco) for 10 minutes at 37°C. Then, the tissue was transferred to a DNase solution containing soybean trypsin inhibitor for gentle mechanical grinding and filtered through a 70-μm cell strainer. The cell suspensions were mixed with Neurobasal medium (Gibco) containing B-27 supplement (Invitrogen), L-glutamine, and penicillin/streptomycin in and seeded in poly-D-lysine-coated 6-well plates at a density of 3-5X10^5^/ml. hUC-MSCs were provided by Yuanpin Cell Biotechnology Group Co., Ltd (http://www.yuanpinbio.com). All the cells were cultured in Dubecco’s Modified Eagle Medium with 10% sterilized fetal bovine serum at 37°C, 90% humidity and 5% CO_2_.

### 2.6 Statistical analysis

The Iba1 positive cells were counted via the spots function of Imaris (Bitplane, INC, UK), which recognized microglia depending on cell diameter. Statistical analyses were performed on GraphPad Prism 9 software (GraphPad Software, San Diego, CA, USA). The difference between the VPA group and the control group was compared using student’s t test, and the treatment groups were analyzed using one-way ANOVA or paired t test. Data were compared as mean ± SEM. Differences between means were regarded as significant at *p <*0. 05.

## 3 Results

### 3.1 hUC-MSCs promoted the growth of primary cultured neurons

**Figure 1:**
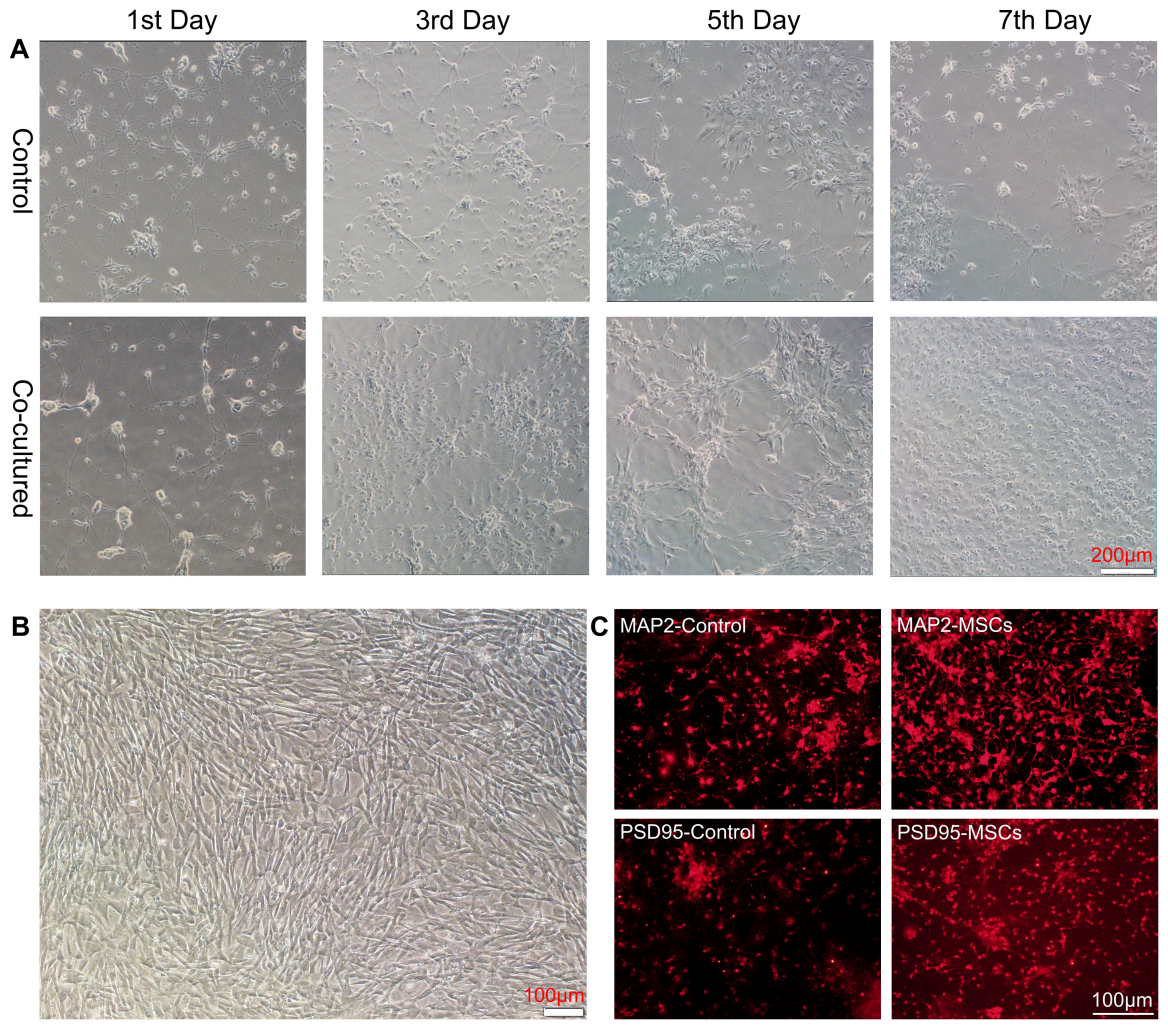
hUC-MSCs promoted the growth and maturation of primary cultured neurons. **(A)** Representative images of primary cultured neurons alone (control, top panel) or co-cultured with hUC-MSCs (Co-cultured, bottom panel) at the 1st, 3rd, 5th and 7th day after plating. **(B)** Representative image of cultured hUC-MSCs. **(C)** Representative immunofluorescence staining of MAP2 (top panel) and PSD95 (bottom panel) in primary cultured neurons from control or co-cultured with hUC-MSCs. Scale bar= 200µm in **(A)**, and 100 µm in **(B, C)**.

To explore whether hUC-MSCs can impact neurons directly, we performed primary neuronal culture from embryos of SD rats and cocultured them with hUC-MSCs using a culture system for transwell migration assay. Neuronal cells grew adherently in the lower layer, and hUC-MSCs were cultured in the upper layer. Two layers were partitioned by a semipermeable membrane made of Matrigel, which allowed medium but not cells to exchange between the layers. Afterwards, the membrane and MSCs were removed, and neural cells were harvested for further testing. As expected, hUC-MSCs growth well in the upper layer (**Figure 1B**), and we observed better growth and longer neurite extension of primary neurons on days 1, 3, 5, and 7 of co-culture (**Figure 1A**). To further reveal the influence caused by hUC-MSCs, we stained the neuronal cells with anti- MAP2 and anti-PSD95, two widely-used markers for mature neurons. The results, depicted in **Figure 1C**, demonstrated that hUC-MSCs greatly enhanced the survival and growth of the neurons. In addition, neurons in hUC-MSCs cocultured group presented higher fluorescence intensity of anti-MAP2 or anti-PSD95 staining compared with the control group (**Figure 1C**). Due to the nature of the transwell migration assay system, we assume that hUC-MSCs may have neurotrophic effects which facilitated the survival and differentiation of primary cultured neurons.

### 3.2 Prenatal exposure to VPA caused tail deformity and aberrant behaviors

**Figure 2:**
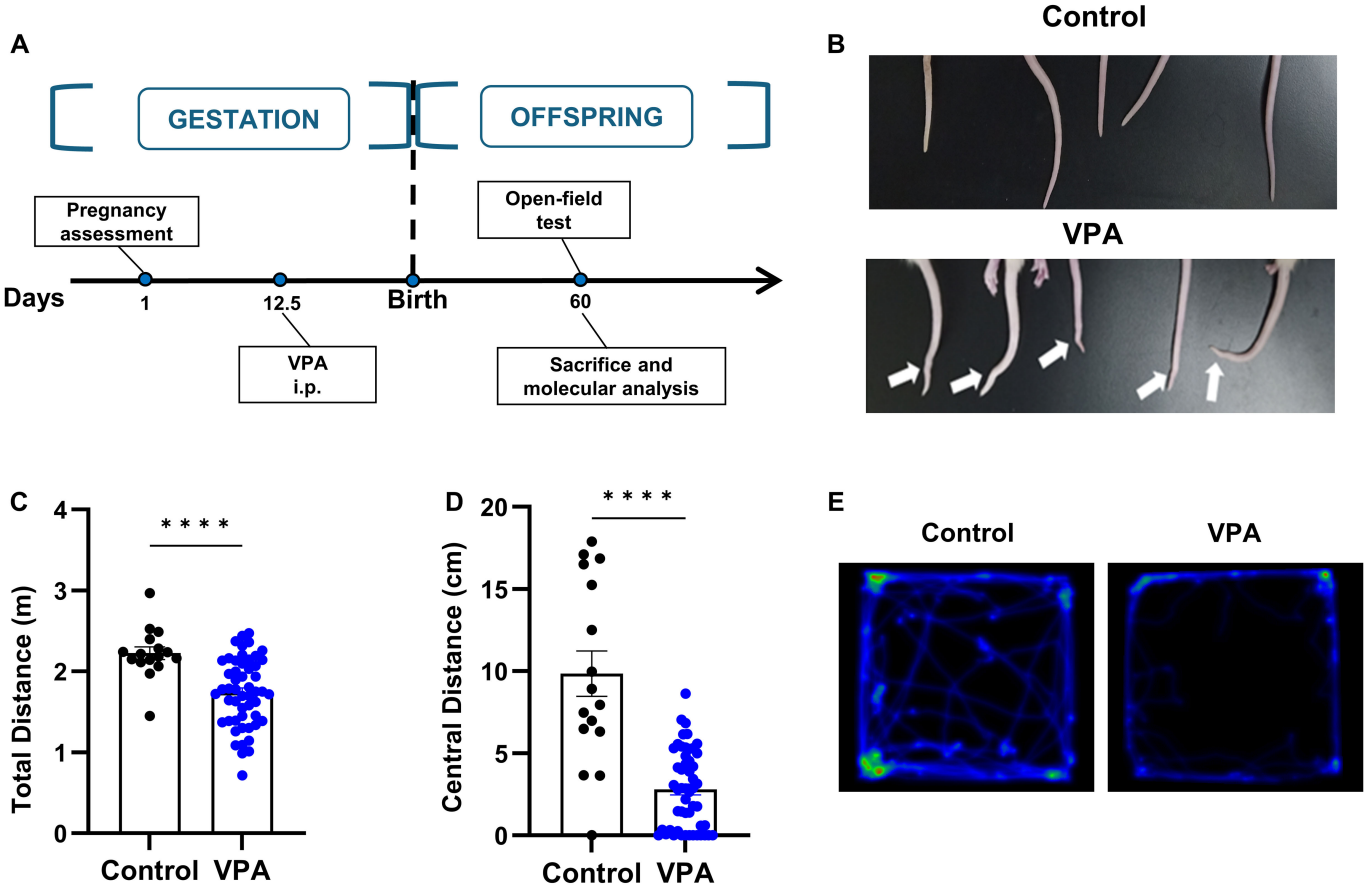
Prenatal exposure to VPA caused tail deformity and behavior disorders. **(A)** Schematic outline of the used protocol. **(B)** Typical tail kick of VPA-exposed offspring. **(C, D)** The total distance **(C)** and central distance **(D)** travelled by the rats in open field test. control: n=16, VPA: n=52. **(E)** Representative hot spot map of the open field test between the control (left) and VPA-treated offspring (right). All data were presented as means ± SEM. Statistical analysis was performed by t test (****p < 0.001).

The VPA-treated animal model has been widely acknowledged and utilized to mimic human autism. Therefore, we conducted preliminary assessment of the face validity of this model using somatic deformity and open field tests (**Figure 2A**). We found that proportions of rat offspring which received a single dose of 400mg/kg of valproate on embryonic day 12.5 presented overt tail kick (**Figure 2B**). We then performed the open field tests and observed a significantly decreased ratio of central distance to total distance traveled by the rats of VPA group (**Figures 2C–E**), indicating augmented anxiety-like behaviors in these rats. In summary, our findings confirm the efficacy of the VPA-induced rat model for studying autism-related behaviors. This validation is essential as it set up the foundation for the subsequent experiments involving hUC-MSC treatments.

### 3.3 Prenatal exposure to VPA induced microglia proliferation

**Figure 3:**
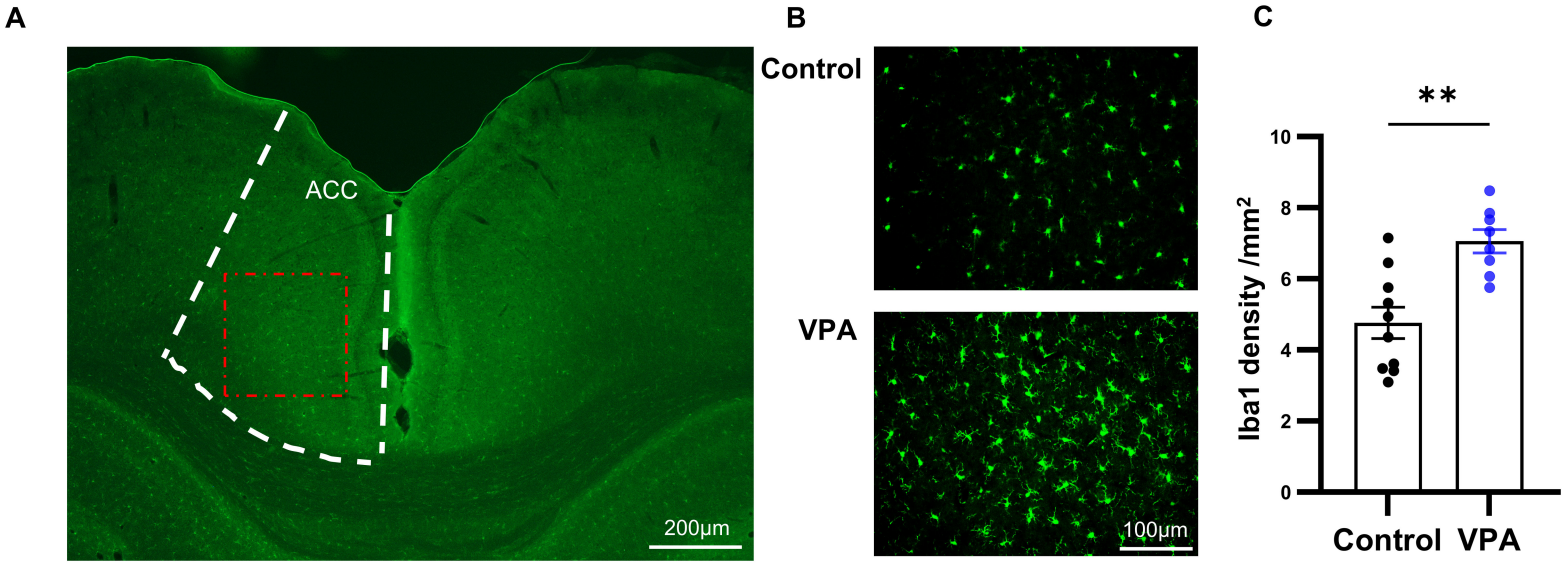
Prenatal exposure to VPA induced microglia proliferation. **(A)** Schematic diagram of the ACC area (boxed by the white broken line). The red dotted box showed the area captured by the microscope and displayed in **(B)** Scale bar= 100µm. **(B)** Representative immunofluorescence images of Iba1 expression in brain sections from control (top) and VPA-treated rats (bottom), **(C)** The number of Iba1 positive cells in control and VPA-treated groups. control: n=10, from 5 rats, Vpa: n=8, from 4 rats. All data are presented as means ± SEM. Statistical analysis was performed by t test (**p < 0.01). Scale bars=200 um in **(A)**, 100µm in **(B)**.

In the next study, we utilized Iba1 staining to quantify the number of microglia in ACC, a crucial brain region implicated in social interaction (23). It is well-established that anterior cingulate cortex is connected with various brain regions involved in social information processing in both humans and rodents. Previous research in autism animal models has demonstrated dysfunction in excitatory and inhibitory synaptic transmission, as well as altered dendritic spine density in ACC (24). Microglia play a pivotal role in the inflammation of the central nervous system. We aimed to investigate the activation of neuroinflammation by quantifying the number of microglia in ACC through immunofluorescence staining of Iba1, a Ca2+-binding protein known for its stable expression in microglia (**Figure 3A**).

Comparing the VPA-exposed group with the control group, we observed a significant increase in the number of Iba1-positive cells in the ACC of the VPA group (**Figures 3B, C**). Furthermore, microglia in the VPA group exhibited characteristics indicative of augmented activity, including enlarged cell bodies and reduced protuberances.

### 3.4 hUC-MSCs treatment relieved VPA-induced social interaction deficiency

**Figure 4:**
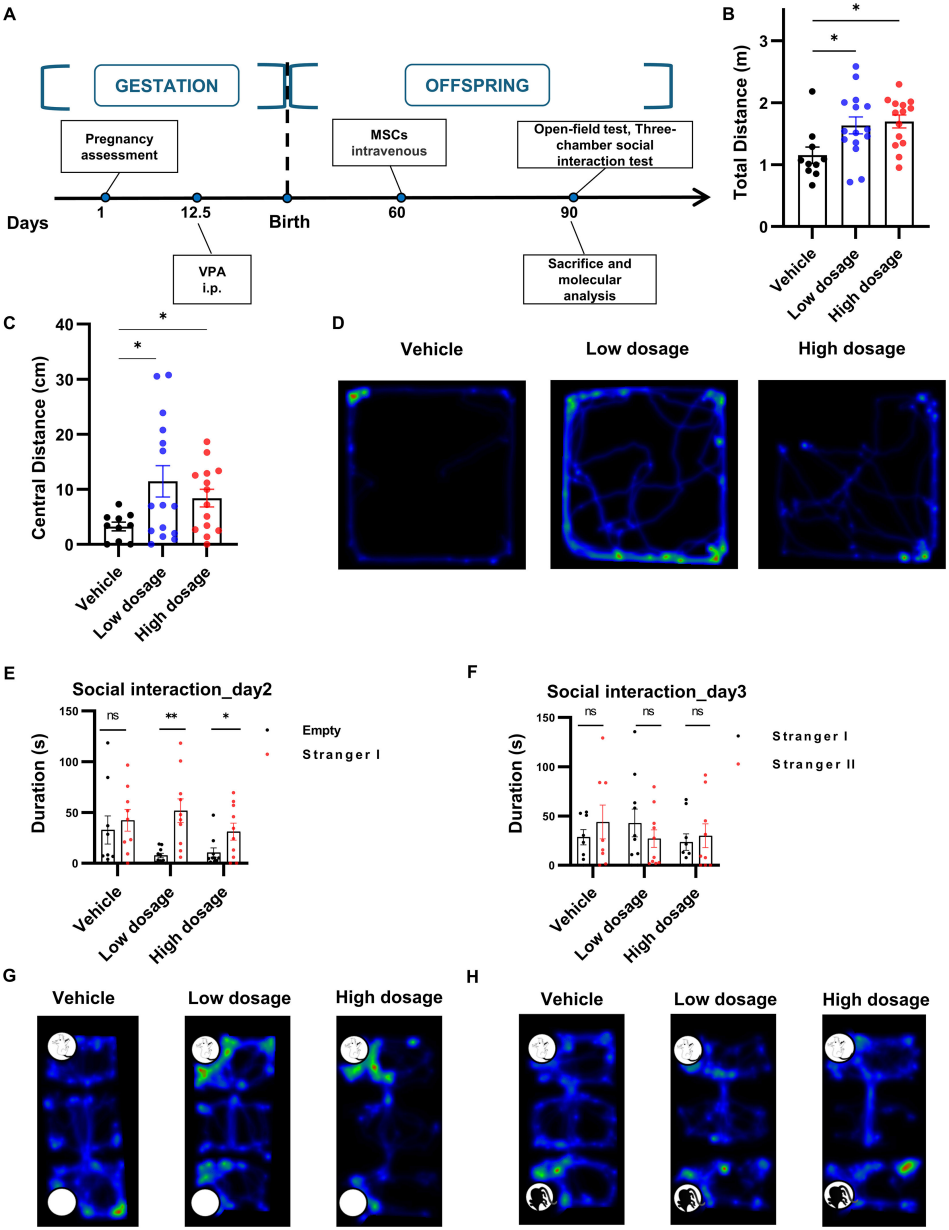
hUC-MSCs treatment relieved VPA-induced social interaction deficiency. **(A)** Schematic representation of the experimental design. **(B, C)** Total **(B)** and central **(C)** distance travelled in open field test, vehicle: n=10, low dosage: n=15, high dosage: n=14. **(D)** Representative hot map of the 3 groups in open field test. **(E, F)** Interaction duration of vehicle, low dosage and high dosage groups in three-chamber social interaction test. **(G, H)** Typical hot map of the three-chamber social interaction test. All data were presented as means ± SEM. Statistical analysis was performed by t test (*p < 0.05; **p < 0.01, ns: no significance).

To ascertain whether hUC-MSCs affected the behaviors of VPA-induced rats, we examined the behavioral manifestation of the three treatment groups in open field and three-chamber social interaction tests after intravenous hUC-MSCs infusion (**Figure 4A**). Surprisingly, as illustrated in **Figures 4B–D**, hUC-MSCs treated rats, regardless of the dosages of hUC-MSCs, displayed significant increase of the distance run in central region as well as the total distance travelled in the open field test, suggesting an improvement in anxiety performance induced by VPA. Similarly, in the three-chamber social interaction test, both groups of hUC-MSCs treated rats, regardless of the dosages again, exhibited strong interest to the 1rt stranger mouse on the 2^nd^ day (**Figures 4E, G**), as evidenced by the significantly increased interaction duration in the compartment where the 1rt stranger mouse was housed (n=9 for vehicle group, n=10 for hUC-MSCs treated groups, p<0.05, paired t test). However, on the 3^rd^ day, no difference was detected among the vehicle group and hUC-MSCs-treated groups (**Figures 4F, H**), showing a potential defect in the social recognition of VPA-treated rats.

### 3.5 hUC-MSCs suppressed VPA-induced microglia proliferation

**Figure 5:**
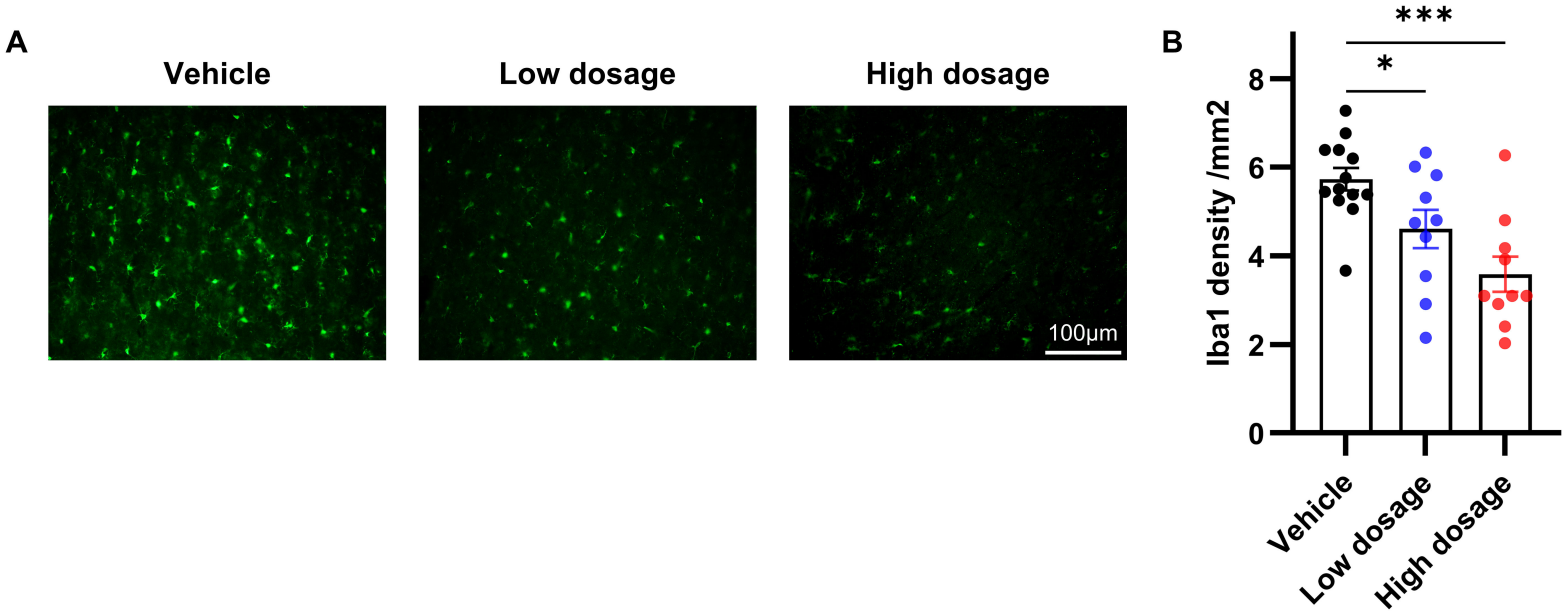
hUC-MSCs suppressed VPA-induced microglia proliferation. **(A)** Representative immunofluorescence images of Iba1 expression in brain sections from vehicle, low dosage and high dosage groups one month later after hUC-MSCs treatment. **(B)** Both the low- and high-dosage groups showed significantly less Iba1-positive cells in ACC. Vehicle: n=13, from 7 rats; low dosage: n=10, from 5 rats; high dosage: n=10, from 5 rats. All data were presented as means ± SEM. Statistical analysis was performed by t test (*p < 0.05; ***p < 0.001). Scale bar: 50µm in **(A)**.

Due to the close association between microglia in the ACC and social symptoms of autism and the immunomodulatory ability of hUC-MSCs, we hypothesized that hUC-MSCs may alleviate social interaction deficits in VPA-treated offspring by targeting the ACC. As depicted in **Figure 5**, we observed an extensive reduction in the number of Iba1-positive cells in both the low and high dosage hUC-MSC-treated groups compared to the vehicle group. Notably, there was no discernible difference or trend in the number of Iba1-positive cells between the low and high dosage MSC-treated groups. In summary, we propose that hUC-MSCs exert the immunomodulatory effects by suppressing the proliferation of microglia which in turn ameliorated the social interaction deficits caused by VPA.

## 4 Discussion

Autism spectrum disorder is a broad range of conditions characterized by challenges with social skills, repetitive behaviors, speech and non-verbal communication (25). Inflammation conditions have long been proposed in ASD, but the precise mechanisms remain incredibly controversial. In this study, we have demonstrated the efficacy of hUC-MSCs in mitigating autism-related symptoms by inhibiting the proliferation of microglia. As a result, hUC-MSCs were able to alleviate behavioral manifestations induced by VPA. Additionally, our findings revealed that hUC-MSCs significantly enhanced neurite growth and extension in a transwell migration assay system, indicating a possible paracrine effect of MSCs on neurons which is independent of glial involvement. Our data highly suggest that hUC-MSCs represent a promising noninvasive treatment approach for ameliorating social interaction deficits in ASD. Our results contribute to a better understanding of the roles played by MSCs in the context of ASD and may pave the way for further research into their therapeutic potential.

It should be noted that the MSCs utilized in this research were obtained from human umbilical cord blood, which was easily accessible and devoid of ethical concerns. To evaluate the potential efficacy of MSCs on rat neurons, we opted in the transwell platform to co-culture hUC-MSCs and rat primary neurons in a contactless manner. Despite the lack of direct interaction, hUC-MSCs enhanced the growth and viability of the primarily cultured neurons. These findings imply that hUC-MSCs can facilitate the growth and survival of neuronal cells through paracrine signaling, and that stem cells can exert their functions across species barriers.

Unlike schizophrenia, bipolar disorder and other mental illnesses, autism has well-established animal model which has been widely utilized in the development of pharmaceuticals and other therapies. Valproic acid is a known teratogenic substance that has been linked to various birth defects including neural tube abnormalities and can result in significant cognitive deficits, delayed language acquisition and diminished social abilities in offspring (26). Increasing evidence has suggested that fetal valproate syndrome (FVS), a condition triggered by exposure to VPA *in utero*, can predispose children with a higher prevalence of autism-related symptoms (26, 27). Schneider and Przewlocki (28) conducted a comprehensive assessment of the offspring behavior of rats exposed to a single dose of VPA on day 12.5 of gestation. Their study revealed that the offspring displayed a notable reduction in social interactions, increased stereotyped behaviors, and deficits in attention. Subsequent research has further validated the potential use of rat offspring as a model for studying autism following exposure to VPA during pregnancy (29, 30). Nowadays, the VPA rat model of autism has become a commonly utilized tool in the investigation of the underlying pathological mechanisms of autism as well as in the development of pharmaceutical treatments (31–33).

One of the most prominent characteristics of ASD is the reduction in social engagement, which is considered as an assessment of treatment effectiveness (34). The three- chamber social interaction experiment is commonly used for assessing changes in social skills in animals with ASD. In this experiment, rats were initially placed in the boxes for acclimatization with empty cages on either side. On the following day, a rat of the same breed and sex was introduced on one side as a ‘social stimulus’, while the other side remains empty as a ‘non-social stimulus’ (35). The test rat was then given the choice to interact with either side. On the third day, a new, unfamiliar rat was placed in the previously empty cage, giving the test rat the opportunity to choose between a familiar or unfamiliar rat companion. The second day of the experiment primarily assesses the social competence of the rats, while the third day provides insights into their social novelty and cognitive abilities. In our experiment, offsprings administered with hUC-MSCs exhibited increased social interaction on the 2^nd^ day as compared to the vehicle group. This suggested that hUC-MSCs effectively mitigated the core symptom associated with reduced social behavior in autism. On the third day, offsprings in the hUC-MSCs treatment group did not show increased social interaction with new unfamiliar mice, indicating that social discrimination abilities in autistic rats treated with hUC-MSCs might still be not improved. In general, hUC-MSCs demonstrate a notable capacity to address dysfunctional social skills associated with autism. However, the enhancement in social novelty remains minimal and further mechanistic studies need to be warranted.

Amounting evidence shows that neuroinflammation is an indispensable mechanism underlying a range of psychiatric disorders such as major depression, ASD, and schizophrenia. Specifically, ASD is recognized as a neurodevelopmental disorder in which microglia play a crucial role (36). Microglia and astrocytes are important in regulating neural circuit function during nervous system development through processes such as synaptic pruning and immunomodulation (37, 38). The role of glial cell dysfunction in ASD has garnered significant attention, with studies demonstrating neuroinflammatory activation in both animal models and clinical populations (39, 40). Clinical trials have shown elevated levels of astrocyte-specific proteins like aquaporin-4 and connexin43 in individuals with autism (41), as well as increased expression of GFAP and astrocyte activation in the cerebellum (42). Additionally, microglial activation and the release of pro-inflammatory factors have been implicated as neuropathological mechanisms in ASD (43, 44).

Bronzuoli et al. have reported that microglia may exhibit heterogeneous roles in the pathology of rat models of ASD induced by VPA treatment (8). They have revealed increased activation of microglia in the prefrontal cortex and hippocampi on postnatal days 35 and 60, with a significant decrease in microglia on postnatal day 90. Thus, it was postulated that microglia contribute more prominently before postnatal day 90, the critical time point corresponding to human adulthood. Additionally, the activation of microglia during infancy and adolescence has a significant impact on the structure and function of dendritic spines on neurons, particularly in excitatory and inhibitory pathways, which is believed to be a contributing factor to ASD pathology. Given the importance of microglia in pathological processes, targeting the excessive activation of microglia may offer a promising therapeutic approach (8). Based on our pilot studies, we chose postnatal day 60 as treatment point and performed the behavioral assessment on postnatal day 90, which may have missed the most sensitive period to modulate microglial function and hence the therapeutic effects of hUC-MSCs may be under-estimated. Nevertheless, since hUC-MSCs were administered on postnatal day 60 and we observed a significant decrease of activated microglia in ACC in the hUC-MSCs-treated group compared to the vehicle-treated group. Similar with prefrontal cortex and hippocampi, ACC has been a typically researched region in the rat models of ASD induced by VPA. Importantly, ACC was considered contributing more to the core symptom of ASD (23). We have robust grounds to conclude that hUC-MSCs exert a discernible effect in the ACC by down-regulating the activation of microglial, thereby alleviating the aberrant behavioral phenotypes presented by rats with VPA-treatment.

In conclusion, our study indicates that hUC-MSCs could potentially enhance neurite growth and extension and reduce abnormal activation of microglia in valproic acid-induced autism model. Importantly, hUC-MSCs can significantly alleviate the main symptoms of social deficits in autism. These results offer valuable insights for clinical translation and further research on the mechanisms of hUC-MSCs in psychiatric disorders characterized by microglial activation, particularly in cases of autism, shall be warranted.

## 5 Conclusion

In this study, we discovered that hUC-MSCs can facilitate neuronal growth and neurite extension as well as ameliorate abnormal microglial activation in autistic brain. These findings offer valuable insights for further research into the mechanisms and hUC-MSCs-based clinical applications in autism treatment.

## Data Availability

The raw data supporting the conclusions of this article will be made available by the authors, without undue reservation.
